# Artificial intelligence-based bi-ventricular systolic and diastolic volume, ejection fraction using non-contrast ECG-gated cardiac computed tomography

**DOI:** 10.1093/ehjimp/qyaf121

**Published:** 2025-10-25

**Authors:** Min-Fang Chao, Athira J Jacob, Abhiraj Sinha, Kristina Hallam, Kristian Hay Kragholm, Puneet Sharma, Saikiran Rapaka, Juan Carlos Ramirez-Giraldo, Su-Min Chang

**Affiliations:** Houston Methodist DeBakey Heart and Vascular Center, Department of Cardiology, Houston Methodist Hospital, Houston, TX 77030, USA; Department of Medical Imaging, Kaohsiung Medical University Hospital, Kaohsiung Medical University, Kaohsiung City 80756, Taiwan; Digital Technology and Innovation, Siemens Healthineers, Princeton, NJ 08540, USA; Houston Methodist DeBakey Heart and Vascular Center, Department of Cardiology, Houston Methodist Hospital, Houston, TX 77030, USA; CT R&D Collaborations, Siemens Healthineers, Malvern, PA 19355, USA; Houston Methodist DeBakey Heart and Vascular Center, Department of Cardiology, Houston Methodist Hospital, Houston, TX 77030, USA; Department of Cardiology, Aalborg University Hospital, Aalborg 9000, Denmark; Digital Technology and Innovation, Siemens Healthineers, Princeton, NJ 08540, USA; CT R&D Collaborations, Siemens Healthineers, Malvern, PA 19355, USA; Digital Technology and Innovation, Siemens Healthineers, Princeton, NJ 08540, USA; Houston Methodist DeBakey Heart and Vascular Center, Department of Cardiology, Houston Methodist Hospital, Houston, TX 77030, USA; Weill Medical College of Cornell University, New York, NY 100, USA

**Keywords:** cardiac CT, non-contrast, bi-ventricular volumes, AI

## Abstract

**Aims:**

Ejection fraction (EF) and end-systolic volume (ESV) are prognostic markers in cardiovascular disease. While MRI provides accurate assessments, its cost limits widespread use. Non-contrast cardiac CT (NCCT), used for coronary artery disease screening, may offer additional functional information. To evaluate the accuracy of AI-derived ventricular volumes and EF from NCCT compared with contrast cardiac CT (CCT) and MRI.

**Methods and results:**

This single center study included 205 patients who underwent cardiac CT for valve planning, divided into retrospective and prospective cohorts. A validated AI algorithm was applied to low-dose NCCT images at end-diastole and end-systole. Right (RV) and left ventricles (LV) volumes and their EFs were compared with CCT and MRI. In the prospective cohort (49 women, 53 men; mean age 73.9 ± 10.3 years), NCCT correlated strongly with CCT for LVEDV (152 mL; –14.2% relative difference; *r* = 0.91) and LVESV (96 mL; +32.6%; *r* = 0.84), with similar correlations for RVEDV (163 mL; –8.4%; *r* = 0.82) and RVESV (121.4 mL; +33.1%; *r* = 0.85). NCCT predicted LVEF <40% with 98% negative predictive value and 87% accuracy. LVEDV correlated strongly with MRI (*n* = 16) for CCT (240 mL; +4.2%; *r* = 0.99) and NCCT (197 mL; –14.3%; *r* = 0.97), as did LVESV for CCT (115 mL; –5%; *r* = 0.99) and NCCT (134 mL; +11%; *r* = 0.97).

**Conclusion:**

AI-derived ventricular volumes from NCCT show moderate to strong correlations, but EF is underestimated. The derived EF can be a screening tool to rule out significant ventricular dysfunction.

## Graphical Abstract

**Graphical Abstract:**
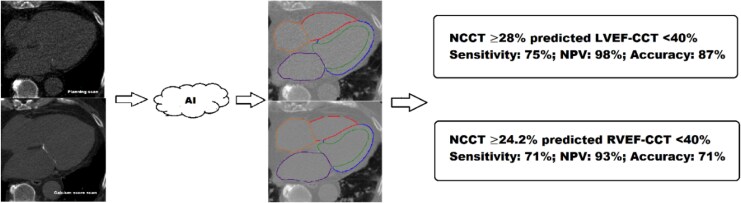

## Introduction

Heart failure (HF) remains a leading cause of cardiovascular morbidity and mortality worldwide, despite significant advancements in therapies and preventive strategies. Left ventricular ejection fraction (LVEF) serves as a key parameter for assessing HF prognosis, with an LVEF below 40% often indicating a terminal phase of the disease and a particularly poor prognosis.^1,2^ Additionally, increased left ventricular end-systolic volume (LVESV) has been associated with adverse events and poor outcomes, emerging as a predictor of HF.^3,4^

Cardiac magnetic resonance imaging (MRI) is the gold standard for quantifying ventricular size, ejection fraction (EF), and stroke volume for both ventricles.^5,6^ However, its high cost, prolonged scanning times, and limited use in patients with implantable devices significantly restrict its accessibility. Echocardiography, while radiation-free, posts challenges as a screening tool for asymptomatic patients due to its labor-intensive nature and limited cost-effectiveness. Additionally, it has difficulty assessing the right ventricle (RV) EF because of the RV's complex anatomy, unique contraction pattern, and positioning within the thorax.^7–9^

Non-contrast cardiac CT (NCCT), commonly used for calcium scoring, offers several advantages, including widespread availability, lower cost, and shorter scan times, making it a screening tool for cardiovascular disease risk stratification and management.^10–12^ While radiation exposure remains a concern, NCCT has gained popularity due to its accessibility and potential to improve long-term outcomes, not only in coronary artery disease risk assessment but also in HF risk evaluation. Recent studies have shown the feasibility of obtaining additional cardiac information from NCCT, including chamber segmentation, particularly in mid-diastole, with good performance.^13–21^

However, no study has yet explored the use of artificial intelligence (AI) algorithms to calculate end-diastolic volume (EDV) and end-systolic volume (ESV) for both the right and left ventricles, and to derive EF as a measure of cardiac function. This study aims to evaluate the feasibility and performance of a validated AI algorithm in calculating EDV and ESV and deriving EF from NCCT scans. These results will be compared with measurements obtained from contrast cardiac CT (CCT) in valve patients to assess the reliability and potential clinical utility of NCCT-based AI algorithms for cardiac function evaluation.

## Methods

### Patients and study design

**Figure 1:**
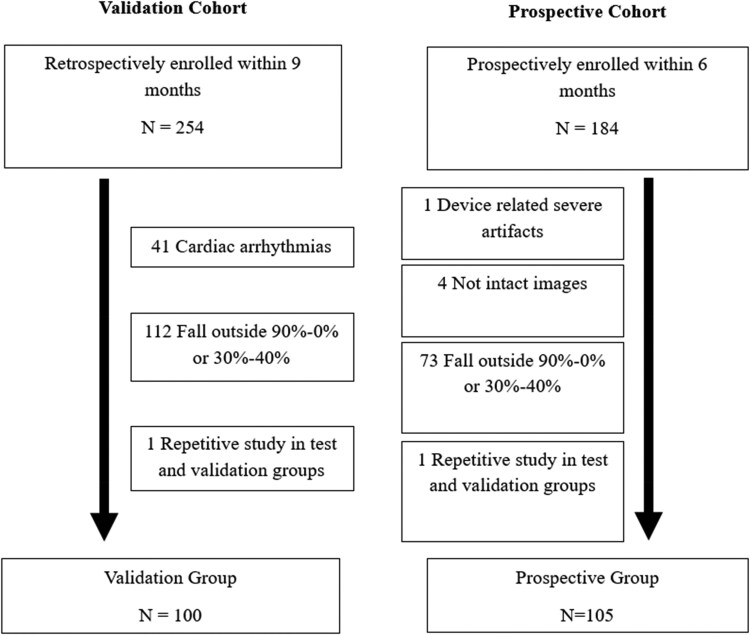
Study design flow chart for validation and prospective cohorts.

This study was conducted at Houston Methodist Hospital in Houston, Texas. The validation cohort consisted of retrospective cardiac CT scans for valve planning collected between June 2023 and February 2024. For the prospective cohort, we prospectively gathered retrospective cardiac CT scans for valve planning between February 2024 and August 2025 (*Figure 1*).

Patients were excluded if they had cardiac arrhythmias, if the scans failed to capture both end-systole (ES) and end-diastole (ED) based on electrocardiogram (ECG) in NCCT studies, if intact images of both ventricles were unavailable, or if the images contained severe artefacts.

Additionally, we collected EF, EDV, and ESV derived from MRI scans performed within 3 months before or after the CT scans, as available in medical records.

This study was approved by the Institutional Review Board (IRB) at Houston Methodist Hospital and adhered to the principles outlined in the Declaration of Helsinki.

### CT protocol and measurement

All cardiac CT scans were performed using a 192-slice dual-source, multi-detector third-generation Siemens SOMATOM Force scanner (Siemens Healthineers, Erlangen, Germany). Two high-pitch, ultra-low-dose, ECG-gated non-contrast scans were acquired using a Tin filter (Sn100 or 120 kVp): one at ED for planning and the other at ES for valve calcium scoring. Image reconstruction parameters were fixed to quantitative medium smooth kernels (Qr36 and Sa36), at 3.0 mm thickness. Retrospective contrast images were reconstructed at 5% intervals with 0.5 mm thickness.

**Figure 2:**
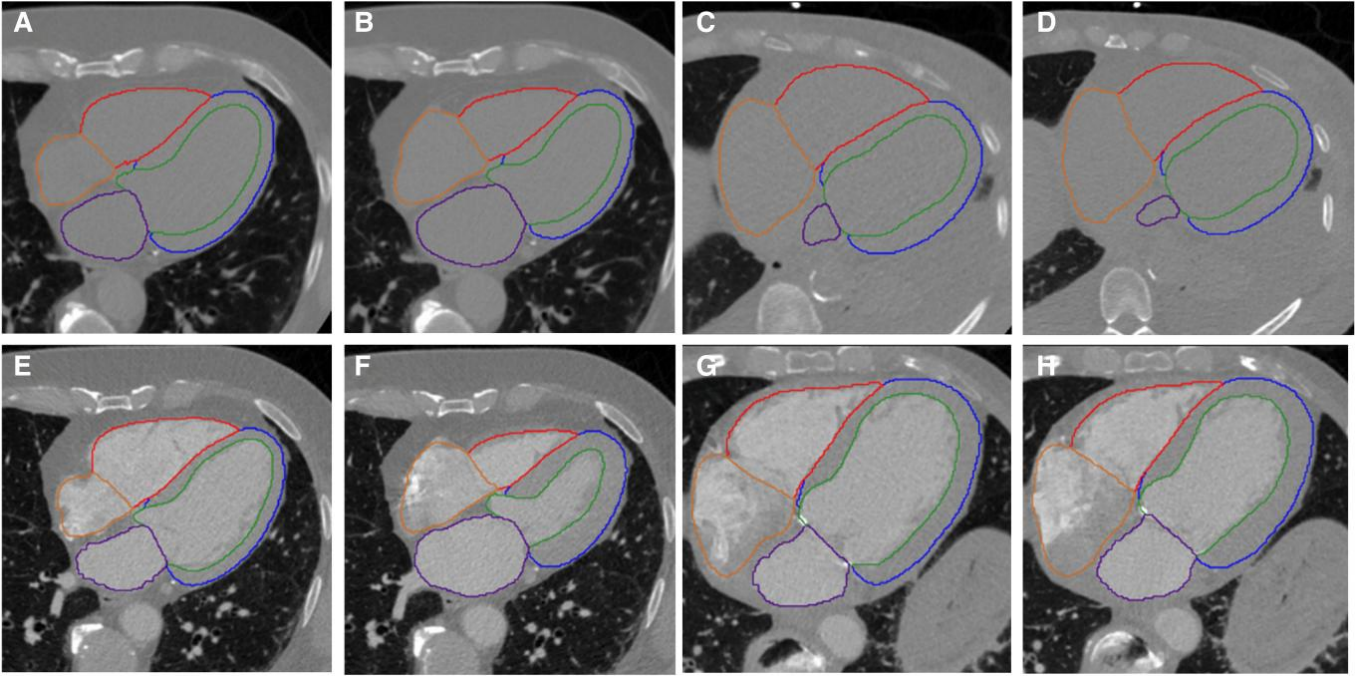
AI-based segmentation of the left and RVs and derived EF. (*A–D*) Chamber segmentation in NCCT, (*E–H*) chamber segmentation in CCT. Panels (*A, B*) and (*E, F*) show a patient with an EF above 60% in CCT, while panels (*C, D*) and (*G, H*) show a patient with an EF below 50% in CCT. NCCT, non-contrast cardiac CT; CCT, contrast cardiac CT.

The contrast images were analysed to calculate the EF using Philips IntelliSpace Portal 9.0 software (Koninklijke Philips Electronics NV, Andover, MA), which served as the ground truth. Additionally, we utilized a previously validated AI-based automatic chamber segmentation algorithm which was trained by NCCT images.^13–15^ LVEF were calculated using NCCT, as shown in *Figure 2*. The EDV was calculated first. The ESV was then determined using the formula: ESV = Total Volume in ESV−Myocardial Volume in ED, assuming the myocardium is incompressible. The LVEF was subsequently calculated as the formula: (EDV – ESV)/EDV. The relative difference in chamber volume was calculated using the following formula: (NCCT– CCT)/CCT.

### Statistical analysis

Patient characteristics were reported as frequencies and proportions for categorical variables and as medians with standard deviations for continuous variables. Group differences were assessed using the χ^2^ test for categorical variables and the *t*-test for continuous variables, as appropriate.

Optimal cut-off points for non-contrast EF in predicting EFs of 40%, 50%, and 60% in the left ventricle based on different scenarios,^1^ and 40% and 50% in the RV, as measured on contrast images, were determined using receiver-operating characteristic (ROC) curve analysis with the Youden index.^22^ Performance was evaluated using sensitivity, specificity, positive and negative predictive values (NPVs), F1 score, and accuracy. In addition, Pearson correlation analysis and Bland–Altman plots were used to assess agreement between methods. We also employed the ANOVA method to compare LVEDV and LVESV values in MRI, NCCT, and CCT.

All analyses were performed using SPSS version 24.0 (IBM SPSS, Chicago, IL, USA) and Python version 3.13. A *P*-value of <0.05 was considered statistically significant.

## Results

In the validation cohort, 254 patients underwent CCTA for valve planning, with 100 enrolled after exclusions. In the prospective cohort, 184 were considered, with 105 included after similar exclusions. Details are in *Figure 1*. Among the 205 total patients, 16 had MRI scans within 3 months of CT.

The basic characteristics of the validation and prospective groups are summarized in *Table 1*. There were no significant differences in age, gender, BMI, or bilateral ventricular volumes between the two groups in NCCT and CT with *P*-value >0.05. All patients (100%) in the validation group underwent retrospective CT for aortic valve evaluation, compared with 99% of patients in the prospective group. The dose–length product showed no significant differences among the validation groups for the planning scan (ED of NCCT), or CCT, but a significant difference was observed for the calcium score scan (ES of NCCT) (*P* < 0.05; Supplementary data online, *Table S1*).

**Table 1:** Basic characteristics of validation and prospective groups

	Validation group	Prospective group	*P*-value
*N*	100	105	
Demographics			
Gender			0.598
Female	43 (43%)	49 (47%)	
Male	57 (57%)	56 (53%)	
Age	74.8 ± 8.9	73.9 ± 10.3	0.506
BMI	28.2 ± 5.2	28.8 ± 6.4	0.474
HR	68 ± 13	72 ± 12	0.033*
Imaging parameters			
Left ventricle			
Non-contrast			
Myocardium (g)	147.7 ± 38.0	152.0 ± 47.3	0.471
LVEDV-NCCT	149.5 ± 50.9	154.6 ± 67.5	0.543
LVESV-NCCT	91.2 ± 44.0	96.0 ± 58.0	0.503
LVEF-NCCT	0.40 ± 0.15	0.40 ± 0.15	0.963
Contrast			
LVEDV-CCT	170.0 ± 57.0	180.2 ± 76.8	0.282
LVESV-CCT	69.6 ± 46.7	72.4 ± 53.9	0.694
LVEF-CCT	0.61 ± 0.14	0.62 ± 0.13	0.900
Right ventricle			
Non-contrast			
RVEDV-NCCT	159.9 ± 47.6	163 ± 64.8	0.703
RVESV-NCCT	116.6 ± 39.9	121.4 ± 59.6	0.499
RVEF-NCCT	0.27 ± 0.10	0.27 ± 0.11	0.795
Contrast			
RVEDV-CCT	173.7 ± 41.2	178.0 ± 53.3	0.521
RVESV-CCT	89.2 ± 32.1	91.2 ± 43.3	0.697
RVEF-CCT	0.49 ± 0.10	0.50 ± 0.09	0.402

BM, body mass index; CCT, contrast cardiac CT; EF, ejection fraction; EDV, end-diastolic volume; ESV, end-systolic volume; HR, heart rate; LV, left ventricle; NCCT, non-contrast cardiac CT; RV, right ventricle.

*P < 0.05.

### Left ventricular volume

**Figure 3:**
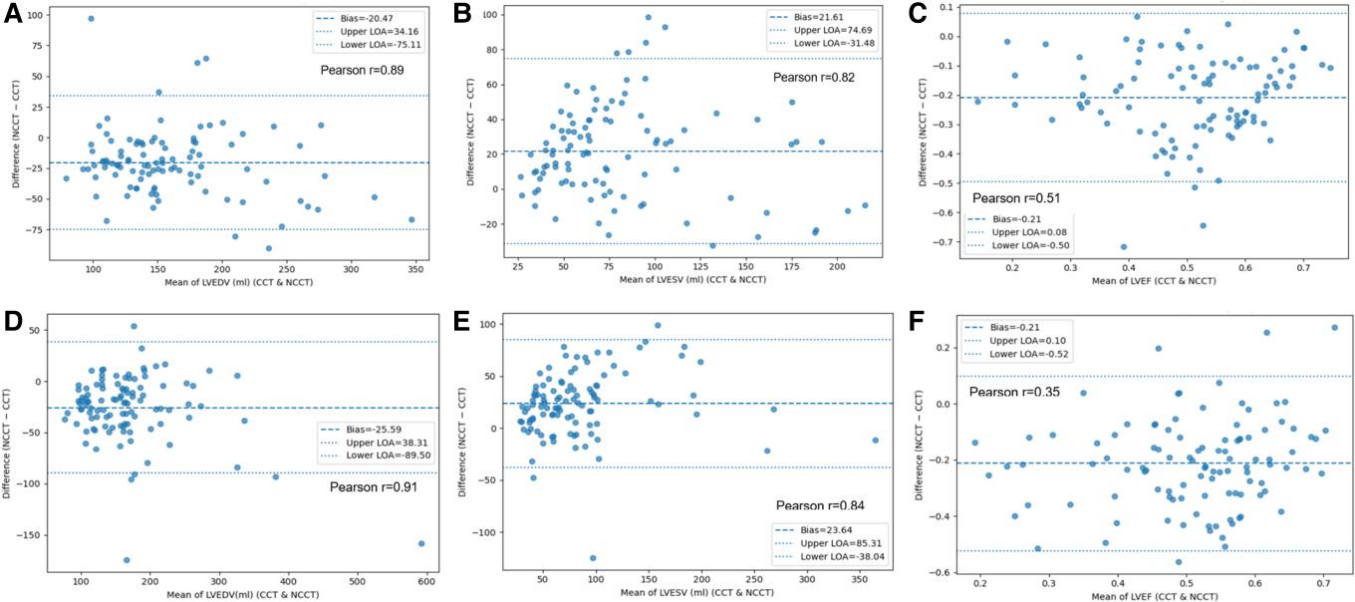
Bland–Altman plots for left ventricular measurements in the validation and prospective cohorts. Bland–Altman plots comparing left ventricular measurements between methods in the validation and prospective cohorts. The solid line indicates the mean bias; dashed lines indicate the limits of agreement (±1.96 SD). Cohorts are shown in separate panels. (*A–C*) The upper panel shows data in the left ventricle from the validation group. (*D–F*) The lower panel represents the prospective group. Panel (*A, D*) represents the end-diastolic volume between NCCT and CCT, panel (*B, E*) shows end-systolic volume, and panel (*C, F*) displays EF.

Strong correlations were observed between NCCT and CCT for left ventricular end-diastolic volume (LVEDV) in both the validation group (NCCT mean: 147.7 mL, –12.0% relative difference compared with CCT; Pearson correlation coefficient *r* = 0.87) and the prospective group (152 mL, –14.2%; *r* = 0.91) (*Figure 3*). Similarly, for LVESV, NCCT averages were 91.2 mL (+31.0% relative difference) in the validation group and 96 mL (+32.6%) in the prospective group, with correlation coefficients of *r* = 0.82 and *r* = 0.84, respectively. Notably, correlations were slightly stronger in the prospective group than in the validation group.

### Left ventricular EF

The correlation coefficients for EF, calculated from LVEDV and LVESV, were relatively low, at 0.35 in the prospective group and 0.51 in the validation group. Optimal NCCT cut-off values for predicting LVEF-CCT were identified as 28%, 40%, and 44.9% for LVEF-CCT thresholds of 40%, 50%, and 60%, respectively, with ROC-AUC values of 0.82, 0.72, and 0.64. In the prospective group, NCCT at a 28% cut-off for predicting LVEF-CCT at 40% demonstrated a sensitivity of 0.75, an NPV of 0.98, and an accuracy of 0.87. For predicting LVEF-CCT at 50%, sensitivity was 0.70, NPV was 0.90, and accuracy was 0.63. At an LVEF-CCT threshold of 60%, accuracy further decreased to 0.57, and NPV dropped to 0.74 (*Figure 6a*).

### Subgroup analysis of cardiac MRI

**Figure 4:**
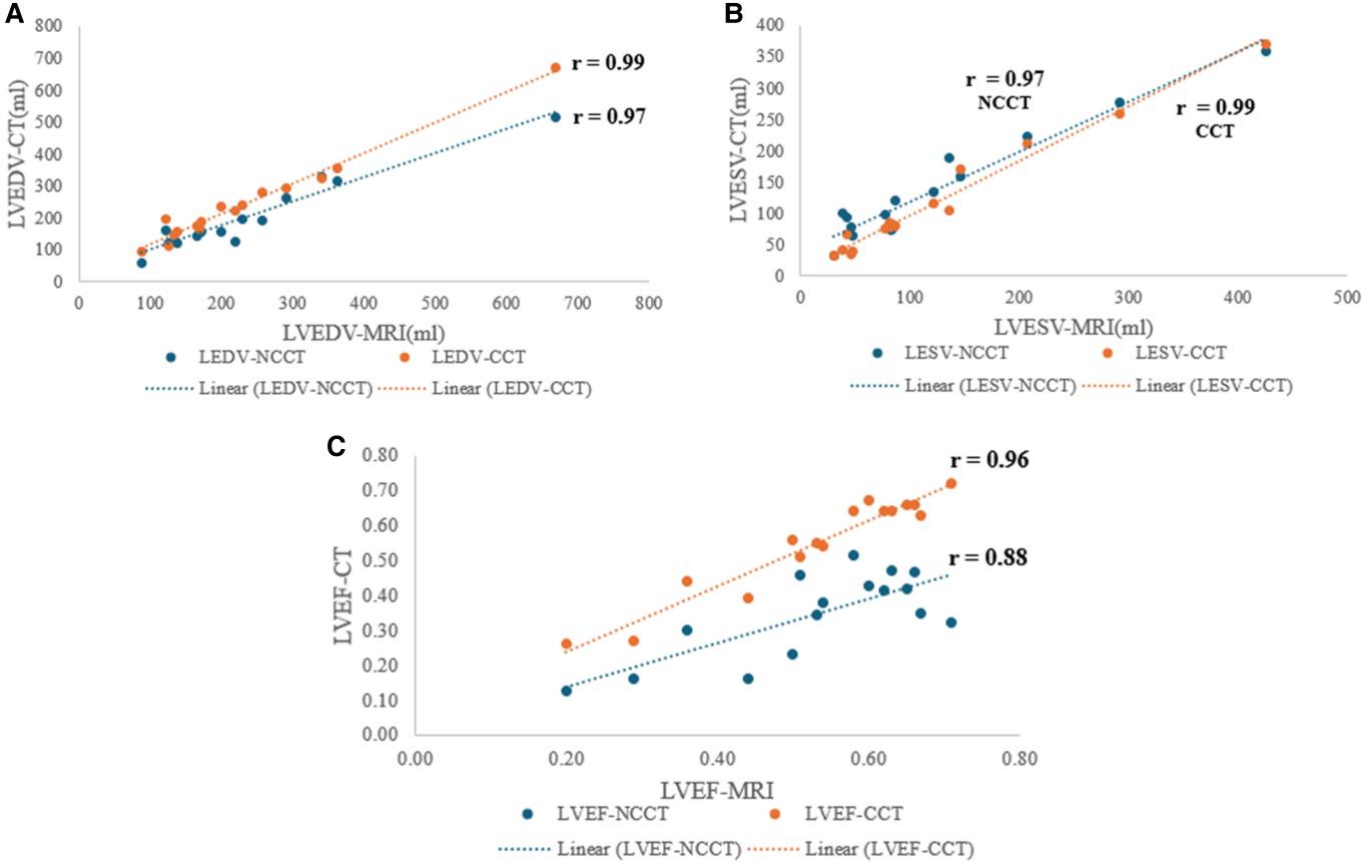
Subgroup analysis of cardiac MRI with NCCT and CCT. (*A*) End-diastolic volume, (*B*) end-systolic volume, and (*C*) EF. NCCT, non-contrast cardiac CT; CCT, contrast cardiac CT.

Among the 205 patients, 16 underwent cardiac MRI scans for comparison. LVEDV measured by CCT (240 mL, +4.2%) and NCCT (197 mL, –14.3%) showed strong correlations with MRI (*r* = 0.99 and *r* = 0.97, respectively). Similarly, LVESV measurements correlated strongly, with CCT (115 mL, –5%) and NCCT (134 mL, +11%) achieving *r* = 0.99 and *r* = 0.96, respectively. However, correlations for EF were slightly lower: *r* = 0.96 for CCT (EF: 0.55, –3.8%) and *r* = 0.88 for NCCT (EF: 0.35, –34%) (*Figure 4*).

### Right ventricular volume

**Figure 5:**
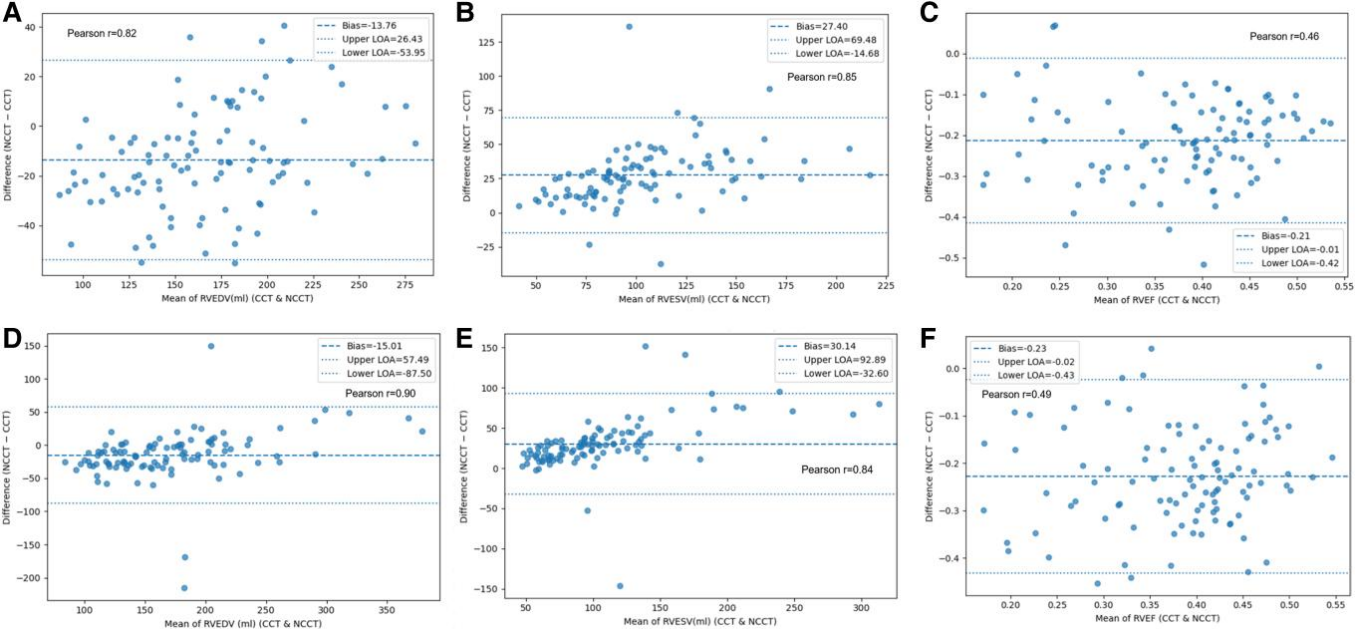
Bland–Altman plots for right ventricular measurements in the validation and prospective cohorts. Bland–Altman plots comparing right ventricular measurements between methods in the validation and prospective cohorts. The solid line indicates the mean bias; dashed lines indicate the limits of agreement (±1.96 SD). Cohorts are shown in separate panels. (*A–C*) The upper panel shows data in the RV from the validation group. (*D–F*) The lower panel represents the prospective group. Panel (*A, D*) represents the end-diastolic volume between NCCT and CCT, panel (*B, E*) shows end-systolic volume, and panel (*C, F*) displays EF.

In the prospective group, strong associations were observed for right ventricular ejection fraction (RVEF), right ventricular end-diastolic volume (RVEDV; NCCT 163 mL, –8.4%), and right ventricular end-systolic volume (RVESV; 121.4 mL, +33.1%). The correlation coefficients for RVEDV and RVESV were 0.82, and 0.85, respectively (*Figure 5*).

### Right ventricular EF

**Figure 6:**
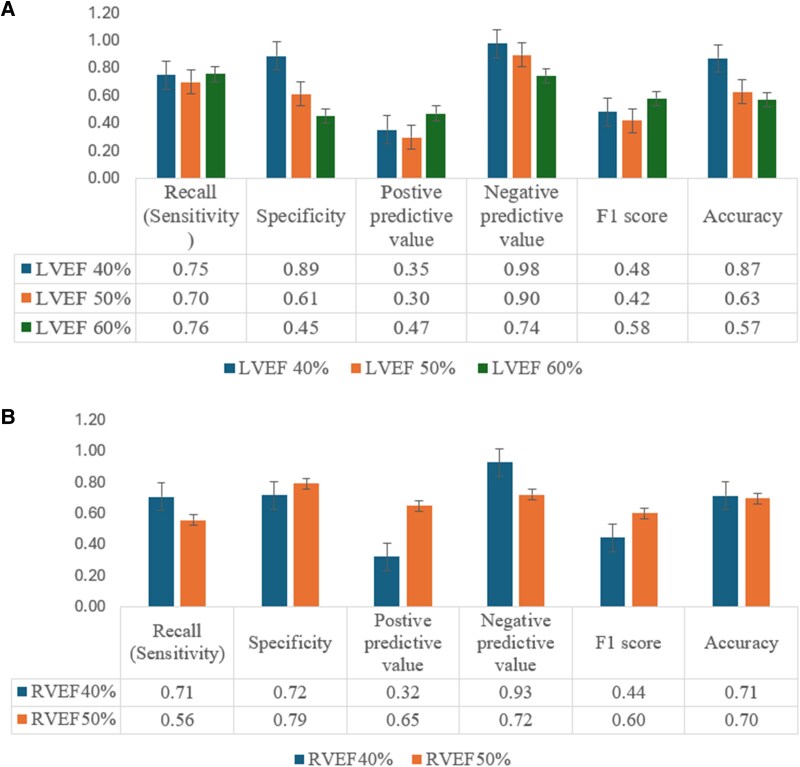
Prediction performance in the prospective group in the bilateral ventricles using different EF standards. (*A*) Upper panel: Performance in the left ventricle using cuff values of 40%, 50%, and 60%. (*B*) Lower panel: Performance in the RV using cuff values of 49% and 50%. LVEF, left ventricular ejection fraction; RVEF, right ventricular ejection fraction.

The correlation coefficient for RVEF was 0.46 in the prospective group and 0.49 in the validation group. The optimal NCCT cut-off value to predict RVEF-CCT thresholds of 40% and 50% was 24.2%, with ROC-AUC values of 0.85 and 0.76. For predicting RVEF-CCT at 40% in the prospective group, sensitivity, NPV, and accuracy were 0.71, 0.93, and 0.71, respectively (*Figure 6b*). At an RVEF-CCT threshold of 50%, the sensitivity, NPV, and accuracy were 0.56, 0.72, and 0.70, respectively.

## Discussion

Our study is the first to apply a validated AI algorithm to measure EDV and ESV across different cardiac cycles using two high-pitch NCCT images and to derive EF, comparing these results with CCT. By adding one NCCT to the standard calcium scoring protocol, the radiation dose remained only about one-fifth of that of a general calcium score scan and substantially lower than that of CCT. This additional NCCT, combined with the original calcium score scan, provided incremental information by enabling EF estimation. We found moderate to strong correlations between NCCT- and CCT-derived chamber volumes for both ventricles. Furthermore, the study demonstrated the feasibility of estimating EF from two NCCT scans, showing high NPV for identifying LVEF and RVEF <40%, comparable to CCT. While the high NPV supports the use of NCCT to rule out severe ventricular dysfunction, precise EF quantification remains less reliable.

### Bi-ventricular chamber volumes

In our study, the AI algorithm was trained using the standard scanning protocol for calcium score studies. We found that the EDV derived from NCCT was significantly smaller—by ∼15% in the left ventricle and 10% in the RV—compared with the values obtained from CCT. Conversely, the ESV was larger, showing a consistent difference of around 32% in both ventricles. This likely resulted from the use of ultra-low dose planning scans for EDV measurements. While some studies report no significant differences in volume between NCCT and CCT when evaluating aneurysmal sacs,^23^ Pim A. de Jong *et al*. suggests that lower radiation doses can lead to edge effects, causing underestimation of size and volume in NCCT compared with CCT.^24^ Phantom studies have also demonstrated that iodine content and material density significantly, phantom size influence error rates. Larger phantoms tend to produce greater errors, with the error rate being lower in medium-sized phantoms compared with smaller or larger ones.^25^ In our study, the use of ultra-low-dose scans with high kilovolt peak (KVP = 120) for AI segmentation may have contributed to the observed differences between NCCT and CCT measurements. Also, in our subgroup analysis with cardiac MRI, we found that the gap between NCCT and CCT measurements increased for small ESV volumes and at the larger end of the EDV range, which aligns with findings from phantom studies. Additionally, while we included patients with scans acquired at two ECG phases—90%–0% and 30%–40%—these phases may not correspond exactly to the optimal ED and ES phases. This is especially relevant for the ES phase, where small variations in the cardiac cycle can significantly affect chamber volumes. Our results also showed greater variation in ESV in both ventricles compared with the EDV.

### Left ventricular chamber volume

In our study, the validated AI algorithm assumes myocardial incompressibility to calculate the LVESV, where the myocardium consists of compacted and non-compacted layers. The non-compacted layer, ∼2.3 times thicker than the compacted layer,^26^ may become more prominent in HF or hypertrophic cardiomyopathy due to thickened papillary muscles, increasing the non-compacted to compacted ratio.^27^ However, since our cohort primarily consisted of patients with severe aortic stenosis and varying degrees of cardiomyopathy, the effect of the compacted myocardium was smaller. As a result, there was a strong association between EDV and ESV for both NCCT and CCT in the left ventricle, leading to an overestimation of the ESV.

### Right ventricular chamber volume

Our study shows that the RV has less variation in EDV but larger variation in ESV compared with the LV in NCCT. Upon reviewing segmentation images, we found that the AI occasionally misclassified parts of the diaphragm or liver dome as part of the RV, particularly in cases of eventration or hepatomegaly. This issue was more evident during the diastolic phase in patients with limited pericardial fat, where the absence of clear fat planes made it difficult to distinguish the heart from adjacent structures as diaphragm or dome of liver on NCCT. In addition, the close anatomical proximity of the RV to the diaphragm and its greater susceptibility to partial volume effects further contributed to segmentation errors and variability in RV measurements. However, these segmentation errors were manually corrected on the CCT images. The study by Goo HW *et al*. also found that the primary cause of segmentation errors in contrast studies was the thin RV wall, especially at the apical portion, which lies flat against the diaphragm.^28^ Additionally, a previous study has shown that lower temporal resolution can lead to an overestimation of RVESV. In our study, we used high-pitch scans with lower temporal resolution, which may have caused significant variation during the ES phase.^29^ Lastly, there is a brief time discrepancy between the ES phases of the RV and LV, as the RV has a longer ejection phase. This could explain the larger variations observed in the RV measurements compared with the LV in our study.

### Bi-ventricular EF

The high correlation observed with EDV and ESV did not extend to EF due to systematic underestimation of EDV and overestimation of ESV, which amplifies errors in EF calculations using the formula (EDV−ESV)/EDV. The underestimation of EDV increases the relative error rate, and when combined with the overestimation of ESV, this discrepancy becomes more pronounced, especially with increasing relative errors in EDV. For instance, in CCT, the LVEDV was 180.2 mL compared with 154.6 mL in NCCT, reflecting a 25.6 mL (14.2%) underestimation of EDV by NCCT. Similarly, the ESV in CCT was 72.4 mL, while NCCT estimated it at 96 mL, resulting in a 23.6 mL (32.6%) overestimation of ESV. These differences significantly affected EF calculations: CCT calculated EF at 59.8%, whereas NCCT calculated it at 37.9%, resulting in a 36.6% relative underestimation. This discrepancy highlights how small errors in EDV and ESV can disproportionately impact EF calculations and explains the better correlation observed with RVEF compared with LVEF in the prospective study. It further emphasizes the need for more refined algorithms to minimize these inaccuracies and improve reliability.

### Performance of EF

#### For left ventricle

The sensitivity of EF derived from NCCT demonstrated consistent performance in predicting LVEF at different thresholds (40%, 50%, and 60%), with sensitivities of 0.75, 0.70, and 0.76, respectively. The NPV remained high at 40% (0.98) and 50% (0.90) but dropped significantly to 0.71 at 60%. These findings suggest that AI algorithms are robust screening tools for ruling out patients with mild or normal ventricular function. We did not exclude patients with moderate pericardial effusion, pneumonia, or prior cardiac surgery, all of which could distort myocardial structure. Despite this, the AI algorithm performed well in identifying patients with LVEF <40%. Notably, in four patients with CCT-measured LVEF <40%, the AI showed discrepancies due to moderate-to-severe pericardial effusion, which likely led to misclassification of the effusion as part of the chamber size, affecting the results.

#### For RV

For the RV, NCCT-derived measurements showed similar performance at EF thresholds of 40% and 50%. However, the correlations for RVEDV and RVESV were lower than for the left ventricle, and the performance of RVEF at 40% was also lower. This is likely due to the AI algorithm not being specifically designed for right ventricular segmentation. Bruns *et al*. found that AI algorithms trained on virtual non-contrast images from CCTA exhibited larger performance gaps when applied to true NCCT images, particularly for the RV. These findings suggest that developing AI algorithms specifically designed for right ventricular segmentation could improve performance and accuracy.

#### Subgroup analysis of cardiac MRI

Although the MRI subgroup was small, we observed strong associations between EF, EDV, and ESV derived from NCCT when compared with both CCT and MRI. Previous studies by Maffei *et al*. and Greupner *et al*. have demonstrated strong agreement between CCT and MRI for both right and left chamber sizes and functions, respectively.^30,31^ As MRI remains the reference standard, our findings should be considered exploratory and interpreted with caution. Larger studies with MRI validation are needed to confirm these results.

## Limitations

Our study has several limitations. First, the AI algorithm was primarily trained on ED phase images and uses an indirect method to calculate LVESV. Second, due to high-pitch scan limitations, we could not accurately capture exact cardiac phases or perform EKG editing. Third, different software platforms for volume calculations in NCCT and CCT may have introduced discrepancies. Despite these, the study showed consistent relative differences of 32% in ESV for both ventricles, indicating the AI algorithm's reliability. Additionally, the small MRI subgroup makes those results exploratory, and the limited sample size dominated by older patients undergoing valve planning for severe aortic stenosis, restricts generalizability to younger populations. Larger, multi-centre studies across different CT platforms are warranted to validate these findings and to establish prognostic correlations for bi-ventricular dysfunction.

## Conclusions

AI algorithms show strong performance in calculating EDV and ESV from two NCCT scans. The derived EF has potential as a screening tool to rule out moderate ventricular dysfunction, with NPVs of 0.98 for LVEF >40% and 0.97 for RVEF >40%. However, it is not precise enough for exact quantification. Further studies are needed to assess correlations with clinical outcomes and to validate their role in patient management.

## Supplementary Material

qyaf121_Supplementary_Data

## Data Availability

No new data were generated or analysed in support of this research.

## Lead Author Biography

Su-min Chang is a board-certified cardiologist with Level 3 training in Echocardiography, Nuclear Cardiology, and Cardiac CT. His research focuses on the clinical application of non-invasive imaging for coronary and structural heart disease. He currently serves as Associate Director of the Cardiac CT Laboratory and Director of Cardiac Imaging at Methodist West Houston Hospital.
