# Supplementary material for: Artificial intelligence-based bi-ventricular systolic and diastolic volume, ejection fraction using non-contrast ECG-gated cardiac computed tomography
Source: Eur Heart J Imaging Methods Pract. 2025 Oct 25;3(4):qyaf121. doi: 10.1093/ehjimp/qyaf121 (PMC12631788; doi:10.1093/ehjimp/qyaf121)
Supplement: qyaf121_Supplementary_Data [file qyaf121_supplementary_data.docx]

| **Supplementary Table 1: Radiation dose of Validation and Prospective groups in NCCT and CCT** | | | |
| --- | --- | --- | --- |
|  | Validation group | Prospective group | P value |
| NCCT radiation dose |  |  |  |
| DLP- Planning scan (mGy.cm) | 19.4+/-15.1 | 19.0+/-14.6 | 0.840 |
| CTDI-Planning scan (mGy) | 0.8+/-0.6 | 0.8+/-0.6 | 0.866 |
| DLP- Calcium score scan (mGy.cm) | 109.8+/-35.0 | 136.7+/-23.6 | <0.001 |
| CTDI-Calcium score scan (mGy) | 6.8+/-2.6 | 10.0+/-1.1 | <0.001 |
| CCT radiation dose |  |  |  |
| DLP (mGy.cm) | 1315+/-559.4 | 1193.8+/-471.6 | <0.001 |
| CTDI (mGy) | 78.3+/-34.1 | 70.3+/-26.7 | 0.057 |
| CCT: contrast cardiac CT; CTDI: Computed Tomography Index; DLP: Dose Length Product; NCCT: Non-contrast cardiac CT.  *p<0.05 | | | |
